# Oxypeucedanin hydrate alleviates rheumatoid arthritis by inhibiting the TLR4-MD2/NF-κB/MAPK signaling axis: Oxypeucedanin hydrate alleviates rheumatoid arthritis

**DOI:** 10.3724/abbs.2024076

**Published:** 2024-05-11

**Authors:** Mengdan Liu, Xueyan Huo, Congcong Li, Yunjie Hu, Haoran Lei, Dong Wang, Lin Zhu, Yucheng Gu, Dale Guo, Lijun Huang, Yun Deng

**Affiliations:** 1 State Key Laboratory of Southwestern Chinese Medicine Resource Chengdu University of Traditional Chinese Medicine Chengdu 611137 China; 2 School of Basic Medical Science Chengdu University of Traditional Chinese Medicine Chengdu 611137 China; 3 School of Medicine Tsinghua University Beijing 100084 China; 4 Syngenta Jealott’s Hill International Research Centre Berkshire RG426EY UK

**Keywords:** oxypeucedanin hydrate, rheumatoid arthritis, TLR4-MD2 complex, NF-κB/MAPK pathway

## Abstract

Rheumatoid arthritis (RA) is an idiopathic and chronic autoimmune disease for which there are currently no effective treatments. Oxypeucedanin hydrate (OXH) is a natural coumarin known for its potent anti-inflammatory properties. However, further investigations are needed to determine its therapeutic efficacy in treating RA. In this study, we evaluate the anti-inflammatory activity of OXH by treating LPS-induced RAW264.7 macrophages. Our results show that OXH treatment reverses the changes in iNOS, COX-2, IL-1β, IL-6, and TNF-α levels. Additionally, OXH reduces ROS production. Further analysis reveals that OXH suppresses the activation of the NF-κB/MAPK pathway. CETSA results show that OXH competes with LPS for binding to the TLR4/MD2 complex. MST experiments demonstrate the specific affinity of OXH for the TLR4/MD2 complex, with a Kd value of 33.7 μM. Molecular docking analysis suggests that OXH binds to the pocket of the TLR4/MD2 complex and interacts with specific amino acids, such as GLY-343, LYS-388, and PHE-345. Molecular dynamics simulations further confirm this conclusion. Finally, we investigate the potential of OXH in treating RA using a collagen-induced arthritis (CIA) model in rats. OXH effectively ameliorates the symptoms of CIA, including improving body weight, reducing swelling and redness, increasing talus volume, and decreasing bone erosion. OXH also decreases the mRNA levels of pro-inflammatory factors in synovial tissue. Transcriptome enrichment analysis and western blot analysis confirm that OXH suppresses the NF-κB/MAPK pathway, which is consistent with our *in vitro* findings.

## Introduction

Rheumatoid arthritis (RA) is a chronic autoimmune inflammatory disease. Although the exact cause remains unknown, it is believed to be affected by both environmental and genetic factors. The primary clinical manifestations of RA include joint injury and synovitis [ 1 , 2]. RA has a global prevalence of approximately 0.1% and is more common in females than in males [3]. The pathogenic mechanism of RA is complicated and is related to bone and cartilage erosion as well as synovial cell fibrosis and proliferation [ 4, 5]. Current clinical treatment options for RA include non-steroidal anti-inflammatory drugs, glucocorticoids, and disease-resistant anti-rheumatic agents. However, these therapeutic agents often cause adverse reactions, including gastrointestinal reactions, organ damage, and allergic reactions [ 6, 7]. Additionally, certain biological agents, such as TNF-α antagonists, may increase the risk of tumor infection [8]. Therefore, there is an urgent need to discover effective compounds with fewer side effects and better outcomes. Traditional Chinese medicine (TCM), with its potential for multiple components, multiple targets, effectiveness, and few side effects, has attracted increasing attention from researchers who are seeking active ingredients [ 9, 10].

According to previous research [11], RA is associated with major pathological features, including synovial hyperplasia and inflammatory infiltration. This occurs when a significant number of immune cells, such as macrophages and T cells, accumulate within synovial tissue and the joint cavity during the development of RA. Due to inflammation, numerous inflammatory factors, such as TNF-α, IL-6 and IL-1β, are released. Simultaneously, these inflammatory factors stimulate synovial cells to produce chemokines, thereby attracting circulating immune cells to the affected site and worsening joint inflammation [12]. Moreover, the inflammatory response further intensifies as synovial cells proliferate in this environment. Therefore, targeting immune cells or synovial cells has emerged as a candidate strategy for treating RA [13] .

The Toll-like receptor 4 and myeloid differentiation factor 2 (TLR4-MD2) complex and its associated pathways are important for inflammation progression [14]. The TLR4-MD2 complex is primarily expressed on the cytoplasmic membrane of immune cells such as monocytes, macrophages, and dendritic cells. It is activated by various substances, such as lipopolysaccharide (LPS) extracted from gram-negative bacteria, intracellular peptides, glycoproteins, and endotoxins [15]. Such activation triggers downstream pathways, such as the NF-κB pathway, leading to inflammation [16].

Oxypeucedanin hydrate (OXH) is a furan coumarin commonly found in various anti-inflammatory herbs, including *Notopterygium incisum*, *Angelica dahurica* [17], and *Whiteflower Hogfennel*. OXH was previously suggested to suppress NO synthase production [18]. However, its effect on treating RA and its underlying mechanism are still unclear.

In the present study, the mechanism of action was explored at both the cellular level using RAW264.7 macrophages and the molecular level using the TLR4-MD2 complex. Furthermore, a collagen-induced arthritis (CIA) model was constructed to investigate the potential of OXH for treating RA.

## Materials and Methods

### Materials

Dulbecco’s modified Eagle’s medium (DMEM) was obtained from Gibco (C11995500BT; Carlsbad, USA). Fetal bovine serum (FBS) was obtained from Excell (FCS500; Suzhou, China). Penicillin-streptomycin solution was obtained from HyClone (SV30010; Logan, USA). Dimethyl sulfoxide (DMSO) was obtained from Gibco (401S032). Phosphate-buffered saline (PBS) was obtained from Boster (AR0030; Wuhan, China). The Cell Counting Kit-8 (CCK8) was manufactured by Baoguang (BG0025; Chongqing, China). Lipopolysaccharide (LPS) was obtained from Beyotime (S1732; Shanghai, China). Total RNA extraction reagent Trizol was obtained from Vazyme (R401-01; Nanjing, China). RT Easy ^TM^ II (Master Premix for First-Strand cDNA Synthesis for Real-Time PCR, RT-01031) and Real-Time PCR Easy ^TM^-SYBR Green I (QP-01012) were obtained from Foregene (Chengdu, China). Mouse IL-6 and TNF-α ELISA kits and rat IL-6 and TNF-α ELISA kits were obtained from 4A Biotech (Beijing, China). The Reactive Oxygen Species Assay kit was obtained from Meilunbio (MA0219-2; Dalian, China). Radioimmunoprecipitation assay buffer (RIPA) was obtained from Beyotime (P0013B). A broad-spectrum protease inhibitor cocktail and broad phosphatase inhibitor were obtained from Boster. The BCA Protein Assay kit was obtained from CWBIO (CW0014S; Beijing, China). Omni-Easy™ Protein Sample Loading Buffer was obtained from Yamei (LT101S; Shanghai, China). Western blot buffer was obtained from Beyotime (P0023B). The PAGE Gel Rapid Preparation kit was obtained from Yamei (PG112). The 180-kDa retained protein marker was obtained from Vazyme (MP102-02). Polyvinylidene fluoride was obtained from Millipore (IEVH07850; Billerica, USA). The Super ECL Plus Western Blotting Substrate was obtained from Biogeound (BG0001; Chongqing, China). The purified recombinant protein complex 10×his-TLR4/MD2 was purchased from R&D Systems (Minneapolis, USA). The His-tag protein labelling kit RED-tris-NTA (MO. L018) and Monolith NT.115 capillaries (MO. K022) were obtained from Nanotemper (Munich, Germany).

### Cell culture

RAW264.7 macrophages were cultured in DMEM supplemented with 10% fetal bovine serum and 1% streptomycin-penicillin antibiotics and incubated at 37 °C under 5% CO _2_. The growth state of the cells was observed. Upon reaching a cell density of approximately 80%, the cells were washed and blown down with complete medium, and continued to be cultured in 6-well plates.

### Cell viability assay

RAW264.7 macrophages (3000 cells/well) in the logarithmic growth phase were cultivated in a 96-well plate. Medium containing OXH at various doses was added following cell adherence to further culture for a period of 48 h. Then, CCK-8 reagent (10 μL) was added for an additional 1 h of incubation. Finally, the cell viability was calculated via enzyme calibration at an absorption wavelength of 450 nm.

### Reverse transcription-polymerase chain reaction (RT-PCR) analysis

RAW264.7 macrophages (100,000 cells/well) in the logarithmic growth phase were cultivated in 6-well plates. After achieving 75% of cell adherence, OXH at various doses was added to the culture for 2 h of incubation. Then, 1 μg/mL LPS was added for 24 h of incubation. Thereafter, 1 mL of Trizol reagent was added to each well for total RNA extraction. Similarly, 1 mL of Trizol was added to the synovial tissue obtained for grinding, after which the supernatant was used for total RNA extraction. The RNA quality and content were assayed using an ultramicrospectrophotometer. To eliminate genomic DNA (gDNA), 5× gDNase Mix was added prior to 5× RO-Easy ^TM^ reverse transcription. Finally, 2.5 μL of cDNA, 5 μL of 2× RT-qPCR EasyTM Mix-SYBR, 1.7 μL of ddH _2_O, and 0.4 μL of the corresponding primers were added for amplification. The sequences of primers used were as follows: *iNOS* (forward: 5′-GAGCCACAGTCCTCTTTGCTA-3′, reverse: 5′-TGTCACCACCAGCAGTAGTTG-3′), *IL-6* (forward: 5′-GGGACTGATGCTGGTGACAAC-3′, reverse: 5′-CAACTCTTTTCTCATTTCCACGA-3′), *TNF-α* (forward: 5′-CCCTCCAGAAAAGACACCATG-3′, reverse: 5′-CACCCCGAAGTTCAGTAGACAG-3’), and *GAPDH* (forward: 5′-GCAAGTTCAACGGCACAG-3′, reverse: 5′-CGCCAGTAGACTCCACGAC-3′). The operating procedures were as follows: 3 min at 95°C, followed by 40 cycles of 10 s at 95°C, 10 s at 59°C and 20 s at 72°C. Once the Ct data had been generated, it was exported and the mRNA expression level of the factor in question was calculated using the 2 ^–ΔΔCt^ method. *GAPDH* was used as a control and the normalized copy number was compared.

### Western blot analysis

RAW264.7 macrophages (100,000 cells/well) in the logarithmic growth phase were cultivated in a 6-well plate. After achieving 75% cell adherence, OXH at various doses was added to the culture for 2 h of incubation, and 1 μg/mL LPS was subsequently added to co-incubate the cells for 24 h. Afterwards, RIPA buffer containing 10% protease inhibitor and phosphatase inhibitor was added for cell lysis. Accordingly, the same protein lysate was added to the synovial tissue obtained for grinding, followed by 15 min of centrifugation at 16,000 *g* to collect the supernatant for detection by BCA Protein Assay kit. Once the total protein concentration was consistent, the 5× instant protein loading buffer was mixed and heated for 10 min at 95°C. Later, the treated protein was separated by 10% sodium dodecyl sulfate-polyacrylamide gel electrophoresis (SDS-PAGE) before being transferred to a PVDF membrane. After 15 min of blocking in rapid blocking solution, the membrane was incubated at 4°C overnight with the corresponding primary antibodies, including anti-TLR4, anti-NF-κB-p65, anti-p-NF-κB-p65, anti-IκB, anti-p-IκB, anti-IKKα/β, anti-p-IKKα/β, anti-p38, anti-p-p38, anti-ERK, anti-p-ERK, anti-JNK, and anti-p-JNK (CST, Boston, USA), as well as anti-COX-2, anti-IL-1β, anti-TNF-α, anti-GAPDH, and anti-tubulin (Proteintech, Chicago, USA), followed by incubation with the corresponding secondary antibodies (HRP-anti-mouse-IgG or HRP-anti-rabbit-IgG; Proteintech) for 2 h at room temperature. Finally, a high-sensitivity enhanced chemiluminescence (ECL) detection kit was used for band visualization, whereas ImageJ was used for band analysis.

### ELISA

RAW264.7 macrophages (100,000 cells/well) in the logarithmic growth phase were cultivated in 6-well plates. After achieving 75% cell adherence, OXH at various concentrations was added to the culture for 2 h of incubation. Later, the cells were further coincubated with 1 μg/mL LPS for 24 h. After 2 h of standing at ambient temperature, the obtained abdominal aortic blood was centrifuged for 10 min at 3000 *g* to obtain the supernatant, which was stored at ‒30°C. Thereafter, ELISA kits were used to measure cytokine levels (TNF-α and IL-6) in both the cell supernatant and rat serum.

### Detection of ROS levels

RAW264.7 macrophages (30,000 cells/well) in the logarithmic growth phase were cultured in a 12-well plate containing a climbing plate. After achieving 75% cell adherence, OXH at various concentrations was added for 2 h of cell treatment, followed by 24 h of co-incubation with 1 μg/mL LPS. Later, 10 μM DCFH-DA contained within the basic medium was added, and the cells were incubated for 30 min at 37°C. After being rinsed with PBS twice, the cells were fixed for 15 min using 5% paraformaldehyde at ambient temperature and subsequently subjected to 8 min of nuclear staining with DAPI. Finally, the slides containing cells were monitored with a fluorescence microscope (Olympus, Tokyo, Japan).

### Cellular thermal shift assay (CETSA)

CETSA was performed to investigate the direct intracellular binding between OXH and TLR4. After 1 h of pretreatment with DMSO or 60 μM OXH, the RAW264.7 cells were washed with PBS containing a protease inhibitor cocktail. Later, the cells were collected, heated for 3 min at different temperatures (35.5, 38.5, 41.7, 44.9, 48.1, 51.3, 54.5, or 57.5°C) and incubated for 3 min at ambient temperature. Subsequently, the cells were lysed with liquid nitrogen for 1 min prior to an additional 3 min of incubation in a metal bath at 30°C. This step was repeated twice. The resulting samples were centrifuged for 20 min at 16,000 *g* and 4°C for protein separation. Finally, western blot analysis was used to analyze the experimental results.

### Determination of compound-protein affinity by a microscale thermophoresis assay

The purified recombinant protein complex 10× His-TLR4/MD2 was dissolved in PBS. Then, the binding affinity between the protein complex and the dye was determined at a Kd value<10 nM. Subsequently, the protein was labelled according to the protocol of the His-tag protein labelling kit RED-tris-NTA. After dissolving the 60 mM tested stock compounds in DMSO, 5% DMSO-PBST buffer was added to the mixture for dilution before the MST assay with a Monolith NT.115 instrument (Nanotemper). Later, 19 μL of the labelled proteins (200 nM) were mixed with 1 μL of the indicated concentrations of compounds in the reaction buffer. MST data were obtained by using 40% infrared laser power and medium-strength MST power. Finally, data analysis and Kd value fitting were performed using Nanotemper MO analysis software.

### Molecular docking

Molecular docking was performed using the Schrödinger suite (2021-3 release). The ligand structure (CAS No. 2643-85-8) was acquired from the PubChem database ( https://pubchem.ncbi.nlm.nih.gov/) and pretreated with the LigPrep module. Meanwhile, the protein TLR4/MD2 complex structure was acquired based on the RCSB database (PDB ID: 3FXI), as previously reported [19]. The crystal structure with the best resolution was selected. Thereafter, hydrogens were added, H-bonds were assigned, water molecules were removed, and protein energy was minimized using the Protein Preparation Wizard module. Furthermore, the SiteMap module was used to identify potential active pockets, and the Receptor Grid Generation module was applied to generate docking grids. Finally, the ligand and protein were docked using the Glide module.

### Molecular dynamics simulation

After docking, the MD simulation was conducted by Gromacs 2018.8 software. The topology file of the protein was obtained by the pdb2gmx command in Gromacs. The RESP charge was calculated by Multiwfn, as previously reported [20]. The file of the ligand was obtained from Sobtop (Tian Lu, Sobtop, Version [dev3.1], http://sobereva.com/soft/Sobtop, accessed 2022-Aug-9). The gmx editconf command was used to set up the system box. The gmx solvate command was used to add water to the system box. The gmx genion command was used to add counterions to the system box. The gmx grompp and gmx mdrun commands were used for energy minimization. After 100 ps of restriction dynamics processing, the gmx grompp and gmx mdrun commands were used for the simulation at 10 ns of conventional dynamics. Finally, the results were checked and displayed using the Xmgrace tool.

### Rat CIA model construction, administration and evaluation

After adaptive feeding for 3 days, 60 male Wistar rats obtained from the SPF Biotechnology (Beijing, China) were subcutaneously injected with 200 μL emulsion that contained 2 mg/mL chicken type II collagen and complete Freund’s adjuvant, whereas the blank group received an equal volume of normal saline. At seven-day intervals, a second immunization was conducted by injecting 100 μL of 2 mg/mL chicken type II collagen and incomplete Freund’s adjuvant subcutaneously into the tail. Rats displaying symptoms of collagen-induced arthritis (CIA) on day 14 were randomized into 3 groups ( *n*=6‒8 each). The final groups included a blank group, a CIA model group, an OXH group (13.5 mg/kg), and a positive control MTX group (13.5 mg/kg). From days 21 to 39, the rats were orally administered saline, OXH, or MTX. The positive control MTX group received two administrations per week, while the blank group received normal saline. Throughout the administration period, the body weight of the rats was determined every 3 days, and the degree of foot swelling was assessed using a Vernier calliper. Additionally, the arthritis indices of the CIA rats were determined based on a visual semiquantitative scoring system. The scores ranged from 0‒4 for every paw, and the maximum score was 16 points. After the rats were sacrificed, their paws, synovial tissue, abdominal aortic blood, spleen, and thymus were collected for subsequent analyses.

### Hematoxylin-eosin (H&E) staining

For each rat, the ankle joint was amputated, the overlying skin and muscle were discarded, and the amputated ankle joint was immersed in a 4% paraformaldehyde solution for 4 h. After being washed three times with PBS and three times with ddH _2_O water, the joint was decalcified through immersion in a decalcification solution with a volume 20 times greater than the joint itself. The decalcification solution was changed on a weekly basis. After 4‒6 weeks, the extent of decalcification was assessed by acupuncture and clamping. Notably, the decalcification was considered complete when no resistance was felt during acupuncture. Thereafter, the tissue was dehydrated, embedded in paraffin, and sectioned. After 4 min of hematoxylin staining and 2 min of eosin staining, the tissue sections were examined.

### Microcomputed tomography (micro-CT) analysis

For each rat, the ankle joint was immersed in 10% formalin for 48 h. After 2 h of wash with PBS and soaking in 75% ethanol, X-ray tomography (PerkinElmer, Waltham, USA) was employed for scanning, and 3D reconstruction was conducted to assess the extent of bone destruction in each rat and calculate the volume of the talus.

### RNA-Seq analysis

Total RNA was extracted from the synovial tissues of the rats, RAW264.7 macrophages, and THP-1 cells in the CIA and OXH groups. Afterwards, RNA-seq was performed using a NovaSeq 6000 sequencer (Illumina, San Diego, USA). The quality of the original data was assessed using the FastQC tool. The clean reads were subsequently aligned to the reference genome using the HISAT2 software.

### Statistical analysis

GraphPad Prism 7.0 software (San Diego, USA) was used for the data analysis. Data are presented as the mean±standard deviation (SD) from three separate replicates of each assay. *P* value less than 0.05 was considered as statistically significant.

## Results

### OXH inhibited inflammation in LPS-induced RAW264.7 macrophages

**Figure 1:**
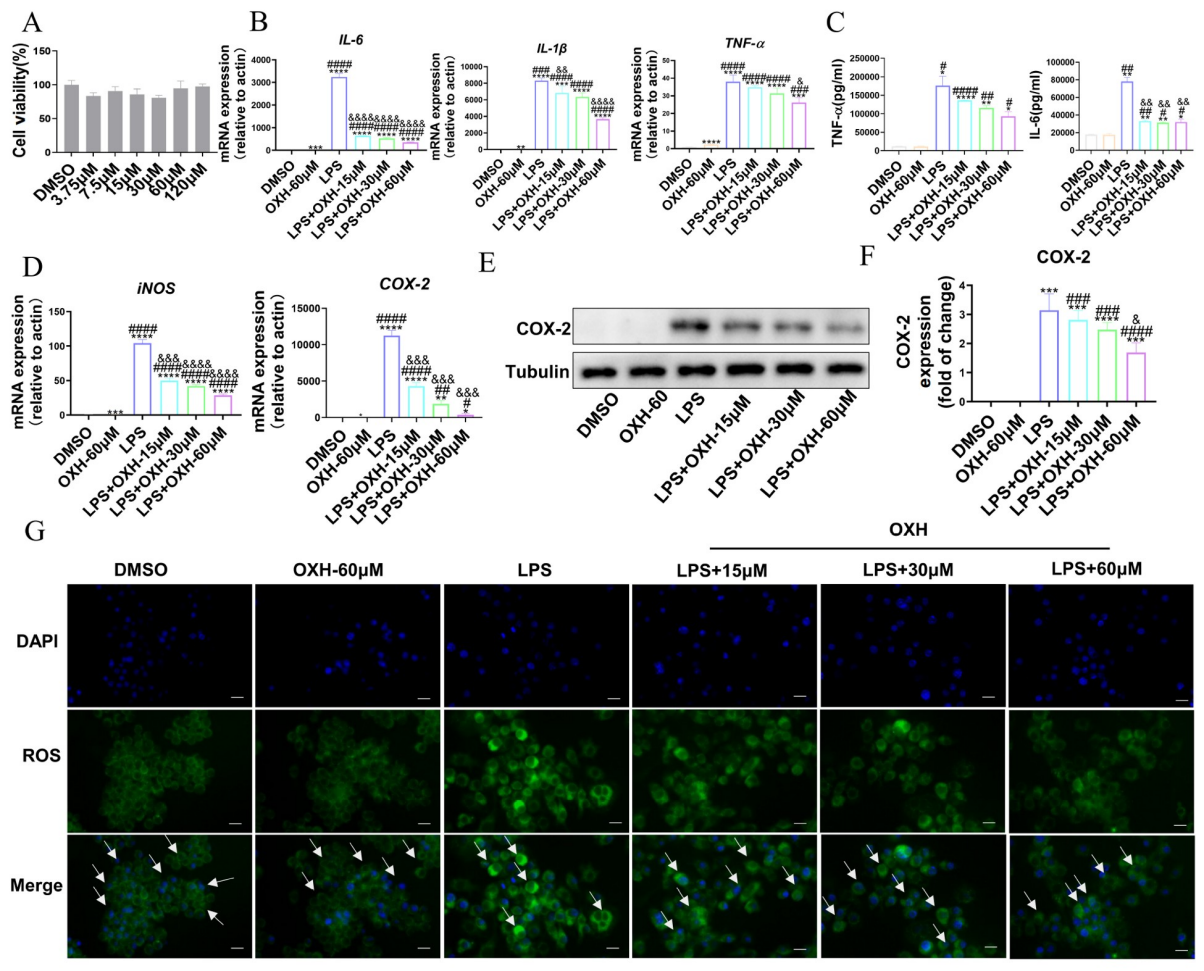
Figure 1 The effect of OXH on the expressions of inflammation-related factors in LPS-induced RAW264.7 macrophages (A) Cell viability was tested using CCK8 reagent after 48 h of treatment with different concentrations of OXH. (B) The mRNA expression levels of IL-6, IL-1β and TNF-α in RAW264.7 macrophages induced by LPS were measured after treatment with different concentrations of OXH. (C) The expression levels of IL-6 and TNF-α in LPS-induced RAW264.7 macrophages were determined by ELISA after treatment with different concentrations of OXH. (D) The mRNA expression levels of iNOS and COX-2 in RAW264.7 macrophages induced by LPS were measured after treatment with different concentrations of OXH. (E) The protein expression of COX-2 in RAW264.7 macrophages induced by LPS and treated with different concentrations of OXH. (F) Quantitative statistical analysis of the protein expression of COX-2. (G) The production of ROS in RAW264.7 macrophages induced by LPS 24 h after OXH treatment. The intracellular ROS were stained with DCFH-DA, with green fluorescence representing ROS and blue fluorescence representing the nucleus. Scale bar: 50 μm. Data are expressed as the mean±SD. *P<0.05, **P<0.01, ***P<0.001, ****P<0.0001, compared with the DMSO group. #P<0.05, ## P<0.01, ###P<0.001, #### P<0.0001, compared with the OXH-60 μM group. & P<0.05, &&P<0.01, &&&P<0.001, &&&& P<0.0001, compared with the LPS group.

LPS-induced RAW264.7 macrophages can stimulate the production of various inflammatory factors, thereby exacerbating inflammation. To verify the function of OXH in suppressing inflammation in RAW264.7 macrophages, we first conducted a cell viability test. OXH did not exhibit toxicity toward RAW264.7 macrophages within a concentration range of 120 μM ( Figure 1A). Subsequent cellular experiments were carried out at concentrations of 15, 30, and 60 μM. The results demonstrated that 15 μM OXH reduced the mRNA expression levels of LPS-induced inflammatory factors, such as *TNF-α*, *IL-6*, and *IL-1β* ( Figure 1B), in a dose-dependent manner, consistent with the ELISA results ( Figure 1 C).

### OXH decreased inflammatory mediator generation in LPS-induced RAW264.7 macrophages

COX-2 overexpression contributes to inflammation-related diseases [21], while increased iNOS enzyme expression can lead to excessive production of NO, which can damage tissues [22]. Additionally, ROS is associated with the activation of signaling molecules related to the production of proinflammatory mediators [23]. Subsequently, the effects of OXH on inflammatory mediators were analyzed. According to the western blot and RT-PCR results, compared with the LPS group, the OXH group exhibited decreased COX-2 and iNOS protein and mRNA levels ( Figure 1D‒F). Furthermore, immunofluorescence analysis demonstrated that the OXH group exhibited lower green fluorescence intensity than the LPS group, suggesting a reduction in ROS production ( Figure 1G).

### OXH blocked the NF-κB/MAPK pathway in RAW264.7 macrophages

**Figure 2:**
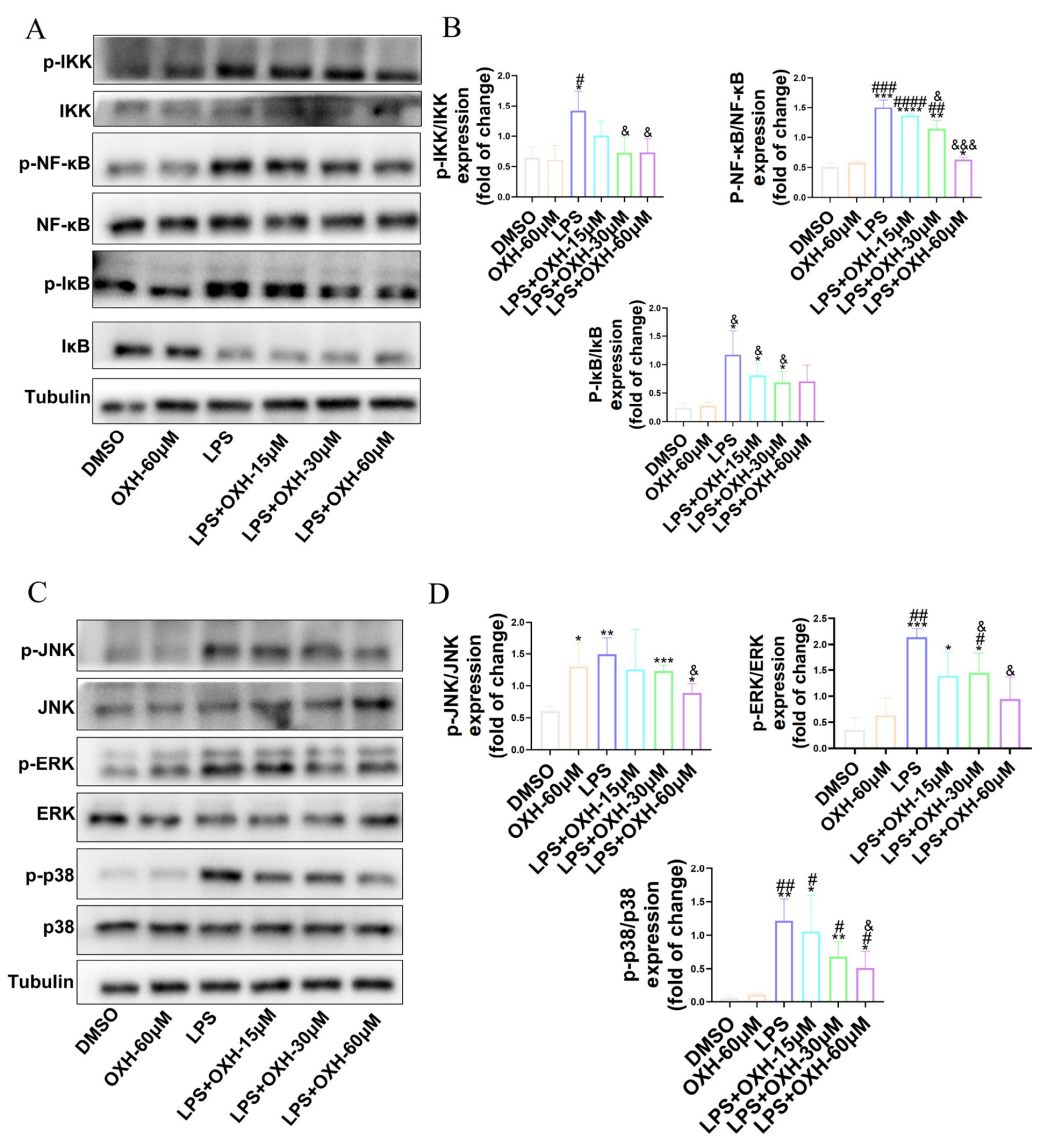
Figure 2 The effects of OXH on the NF-κB/MAPK pathway in LPS-induced RAW264.7 macrophages (A) The protein expression levels of p-IKK, IKK, p-NF-κB, NF-κB, p-IκB, and IκB were analyzed in RAW264.7 macrophages induced by LPS and treated with different concentrations of OXH. (B) Quantitative statistical results of the protein expression levels of p-IKK/IKK, p-NF-κB/NF-κB, and p-IκB/IκB. (C) The protein expression levels of p-JNK, JNK, p-ERK, ERK, p-p38, and p38 were analyzed in RAW264.7 macrophages induced by LPS and treated with different concentrations of OXH. (D) Quantitative statistical analysis of the p-JNK/JNK, p-ERK/ERK, and p-p38/p38 protein expression levels. Data are expressed as the mean ±SD. *P<0.05, **P<0.01, ***P<0.001, ****P<0.0001, compared with the DMSO group. #P<0.05, ##P <0.01, ###P<0.001, ####P<0.0001, compared with the OXH-60 μM group. &P <0.05, &&&P<0.001, compared with the LPS group.

To investigate the potential function of OXH in suppressing the NF-κB/MAPK pathway in RAW264.7 macrophages, western blot analysis experiments were conducted. The p-NF-κB/NF-κB, p-IKK/IKK, p-IκB/IκB, p-ERK/ERK, p-p38/p38, and p-JNK/JNK protein levels were downregulated after OXH treatment compared with those in the LPS group ( Figure 2A‒D). Based on the above results, OXH may inhibit inflammation by blocking the NF-κB/MAPK pathway.

### CETSA revealed that OXH bound to TLR4

**Figure 3:**
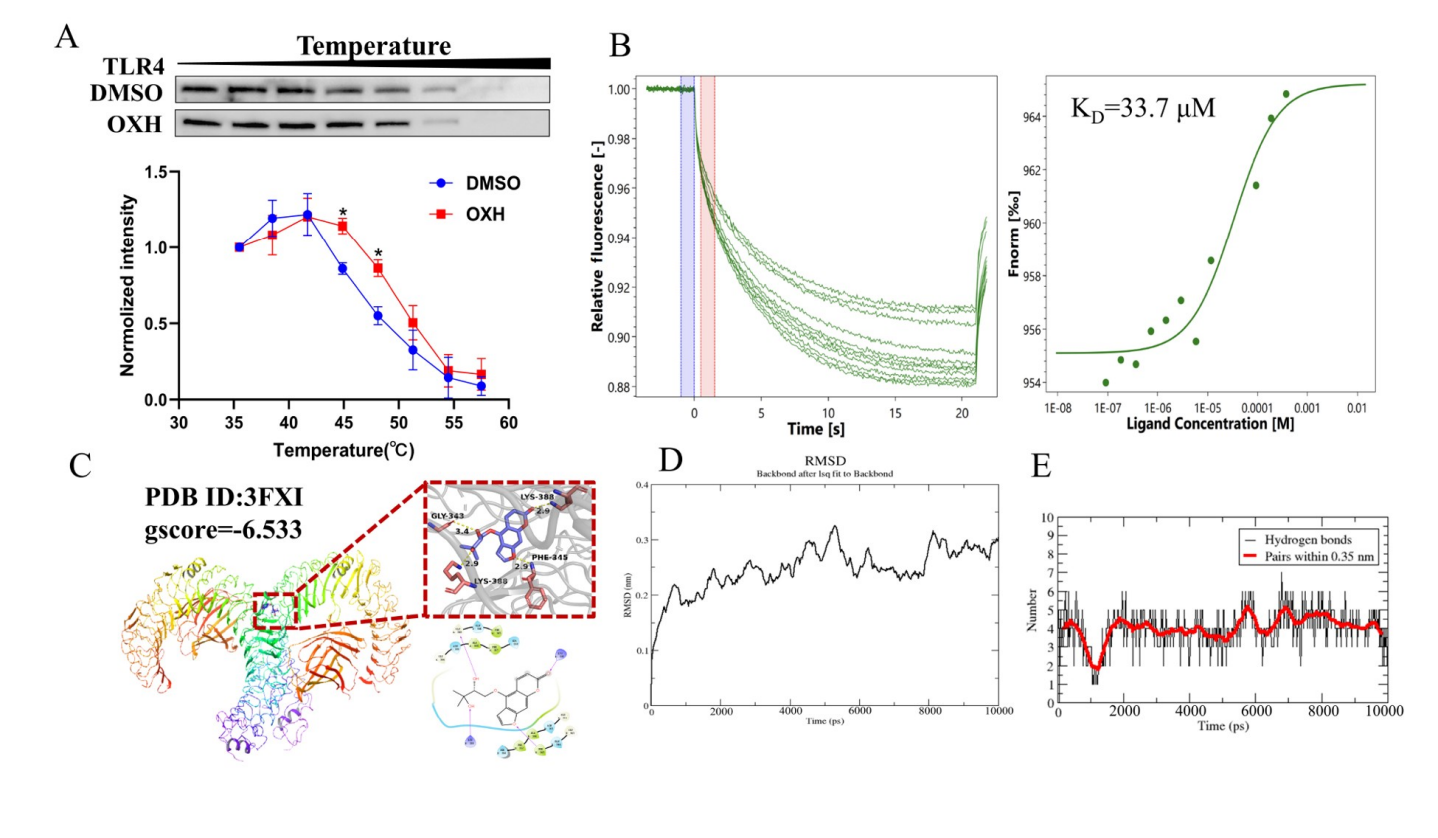
Figure 3 Identification of OXH as a direct target of TLR4/MD2 (A) Evaluation of the CETSA melt response. RAW264.7 macrophages were subjected to treatment with either DMSO or OXH, followed by exposure to varying temperatures. Subsequently, the cells were collected for western blot analysis using an anti-TLR4 antibody. (B) The interaction between OXH and TLR4/MD2 was demonstrated through MST assay. (C) Molecular docking results revealing the binding of OXH to TLR4/MD2. (D) Root mean square deviation of proteins and ligands over time. (E) Analysis of hydrogen bonds between proteins and ligands.

As the receptor of LPS, TLR4 is important for inflammation both *in vitro* and *in vivo*, and we also examined the binding of OXH to TLR4 through the CETSA method. TLR4 expression in the DMSO-treated group decreased from 44.9°C onwards. However, after OXH treatment, TLR4 was still detectable at temperatures up to 48.1°C ( Figure 3A). The CETSA results demonstrated that OXH interacts with TLR4 and enhances its structural stability.

### Identification of TLR4/MD2 as a direct target of OXH

Subsequently, we conducted MST analysis and molecular docking assays to investigate the potential binding of OXH to the TLR4/MD2 complex. First, MST analysis was conducted to validate the interaction of OXH with the TLR4/MD2 protein complex. As shown in Figure 3B, the affinity of OXH for the TLR4/MD2 proteins was remarkable, and the K _D_ value was 34.07±1.00 μM, as determined by MST. Furthermore, according to the molecular docking results, OXH effectively docked with the pocket of TLR4/MD2 and interacted with several amino acids, such as GLY-343, LYS-388, PHE-345 and LYS-388, on another strand ( Figure 3C). The OXH-TLR4/MD2 interaction was further confirmed by molecular dynamics simulation. The conformational stability of TLR4/MD2 and OXH was assessed from the root mean square deviation (RMSD) plot. The system of OXH and TLR4/MD2 reached a balance at 2000 ps, with a stability of 0.25 ( Figure 3D). The results of the hydrogen bond analysis showed that OXH and TLR4/MD2 formed an average of approximately 4 hydrogen bonds per frame after equilibrium was reached in the simulation ( Figure 3E). This result is consistent with the molecular docking results. Taken together, these findings revealed that OXH directly binds to the TLR4/MD2 complex, while subsequent allosteric regulation may inhibit LSP from binding to this complex. Therefore, OXH has the potential to suppress the NF-κB/MAPK pathway by binding to the TLR4/MD2 complex.

### Oral OXH protected against collagen-induced arthritis

**Figure 4:**
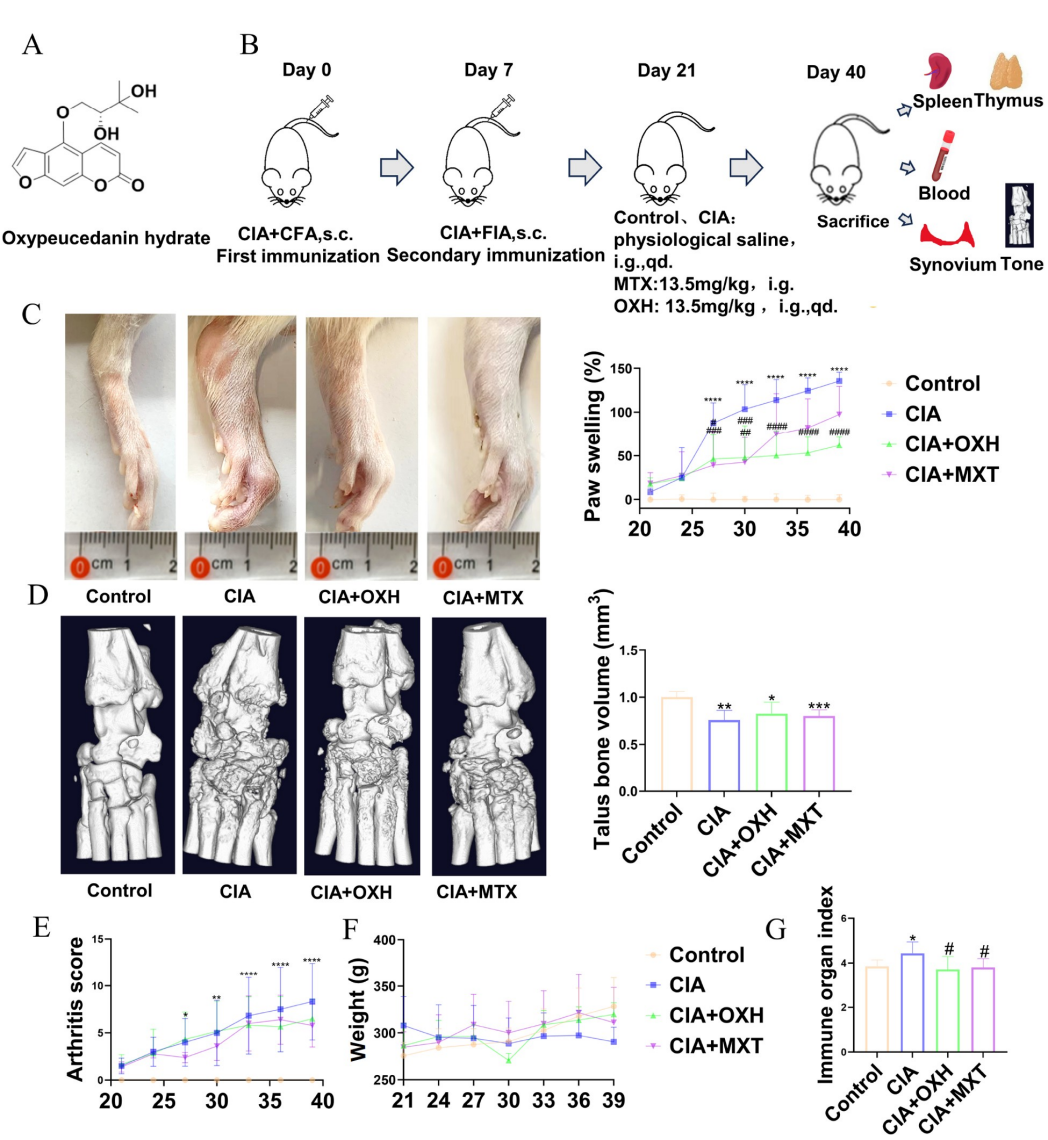
Figure 4 Changes in symptoms after treatment with OXH in a rat model of RA (A) The structure of OXH. (B) The experimental procedures for RA rat modelling, administration, and observation of phenotypic changes. (C) After 19 days of administration with OXH and the positive control drug methotrexate to the RA rat model, foot swelling in each group of rats was observed. The degree of foot swelling in each group was measured using a Vernier calliper every 3 days after administration, and the results are presented in the statistical analysis chart. (D) CT scans were used to observe the bone erosion damage caused by OXH and methotrexate in the RA rat model, and the volume of the talus bone in each group was analyzed statistically. (E) The effect of OXH on the arthritis score at different time points. (F) The effect of OXH on body weight at different time points. (G) Statistical analysis of the spleen and thymus indices in each group. Data are expressed as the mean±SD. *P<0.05, **P<0.01, ***P<0.001, compared with the control group. # P<0.05, compared with the CIA group.

**Figure 5:**
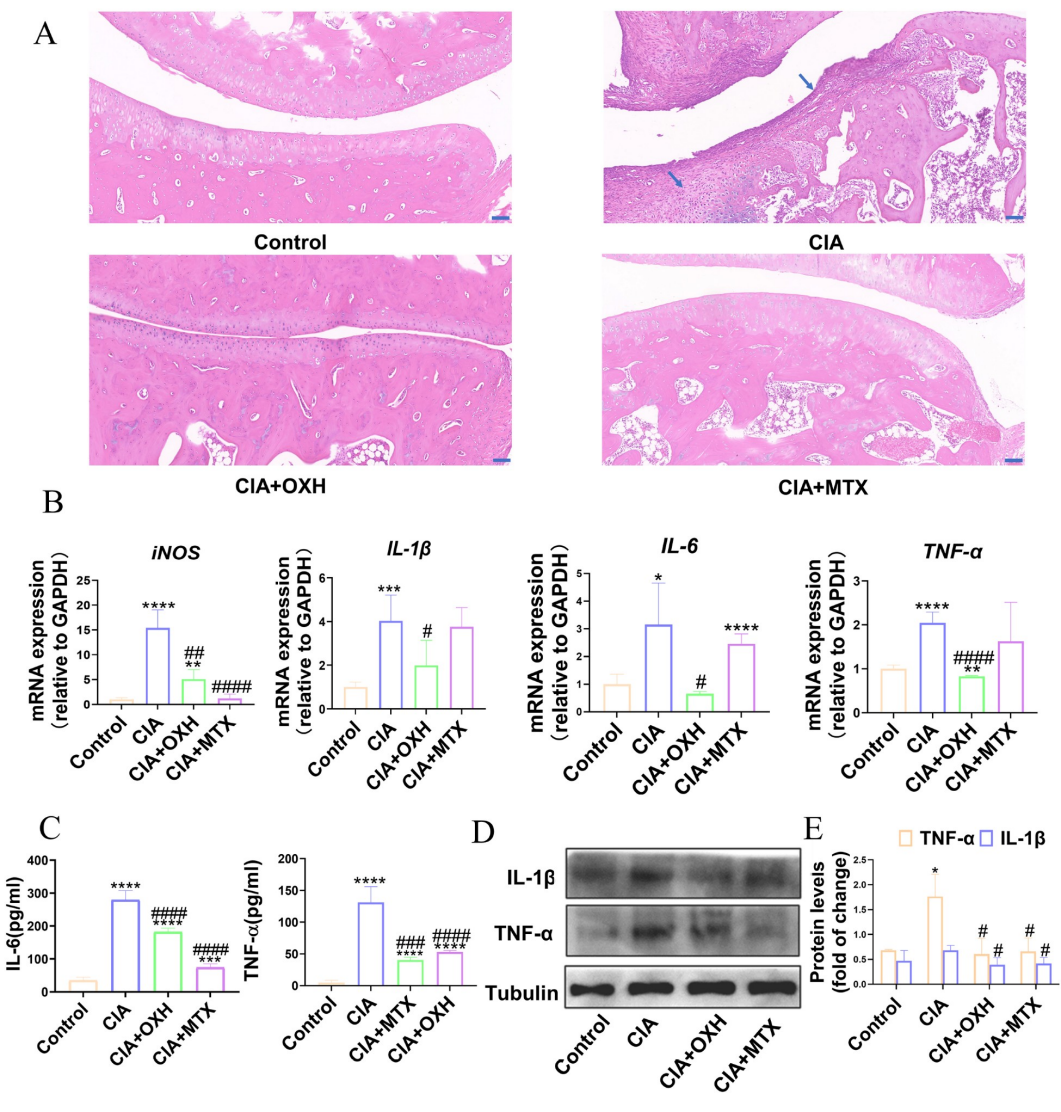
Figure 5 The effects of OXH on the gene and protein expressions of inflammation-related factors in a CIA model (A) Synovial cells in each group were stained with H&E (scale bar: 10 μm). (B) The mRNA expression levels of iNOS, IL-6, IL-1β, and TNF-α in the synovial cells of rats from different treatment groups. (C) The protein levels of IL-6 and TNF-α in the serum of rats in different treatment groups detected by ELISA. (D) The protein expressions of IL-1β and TNF-α in synovial cells from different treatment groups were analyzed, with tubulin serving as an internal reference. (E) Quantitative analysis of the protein expressions of IL-1β and TNF-α. Data are expressed as the mean±SD. * P<0.05, **P<0.01, ***P<0.001, ****P<0.0001, compared with the control group. # P<0.05, ##P<0.01, ### P<0.001, ####P<0.0001, compared with the CIA group.

To investigate the function of OXH in RA, a Wister rat model of CIA was generated. On the 21st day, the CIA rats showed noticeable swelling and redness. Later, the rats were orally administered OXH and methotrexate, while the blank group received water orally from the 21st to the 40th day ( Figure 4A,B). The results indicated significant changes in the arthritis index scores, swelling, redness, and body weight of CIA model rats relative to those of normal rats. However, OXH administration improved the severity of arthritis in the rats ( Figure 4C,E,F). Additionally, we measured the weights of the thymus and spleen of each rat and observed a partial restoration of the immune organ index ( Figure 4G). Three-dimensional CT revealed a decrease in talus volume and severe bone erosion in the CIA group. Remarkably, after OXH treatment, the talar volume increased, and bone erosion decreased ( Figure 4D). Moreover, H&E staining revealed tissue necrosis, articular cartilage fibrosis, chondrocyte proliferation, disordered arrangement, and disappearance of the tidal line in the CIA group. The synovial tissue exhibited localized joint fibrosis and a small amount of proliferation ( Figure 5A). In conclusion, OXH has the potential to alleviate symptoms in CIA rats.

### The effect of OXH on CIA-related inflammatory factors

Previous studies have demonstrated that RA leads to immune cell infiltration and an increase in inflammatory factors, which in turn worsens RA symptoms [24]. The levels of proinflammatory factors such as TNF-α, IL-1β, and IL-6 were measured by ELISA and western blot analysis. Figure 5C,D showed that TNF-α, IL-1β and IL-6 expressions were significantly decreased in both serum and synovial tissue. These results were consistent with the mRNA expression levels illustrated in Figure 5B. These findings suggested that OXH inhibited the inflammatory response in CIA rats.

### Exploration of OXH-related mechanisms via transcriptomics

**Figure 6:**
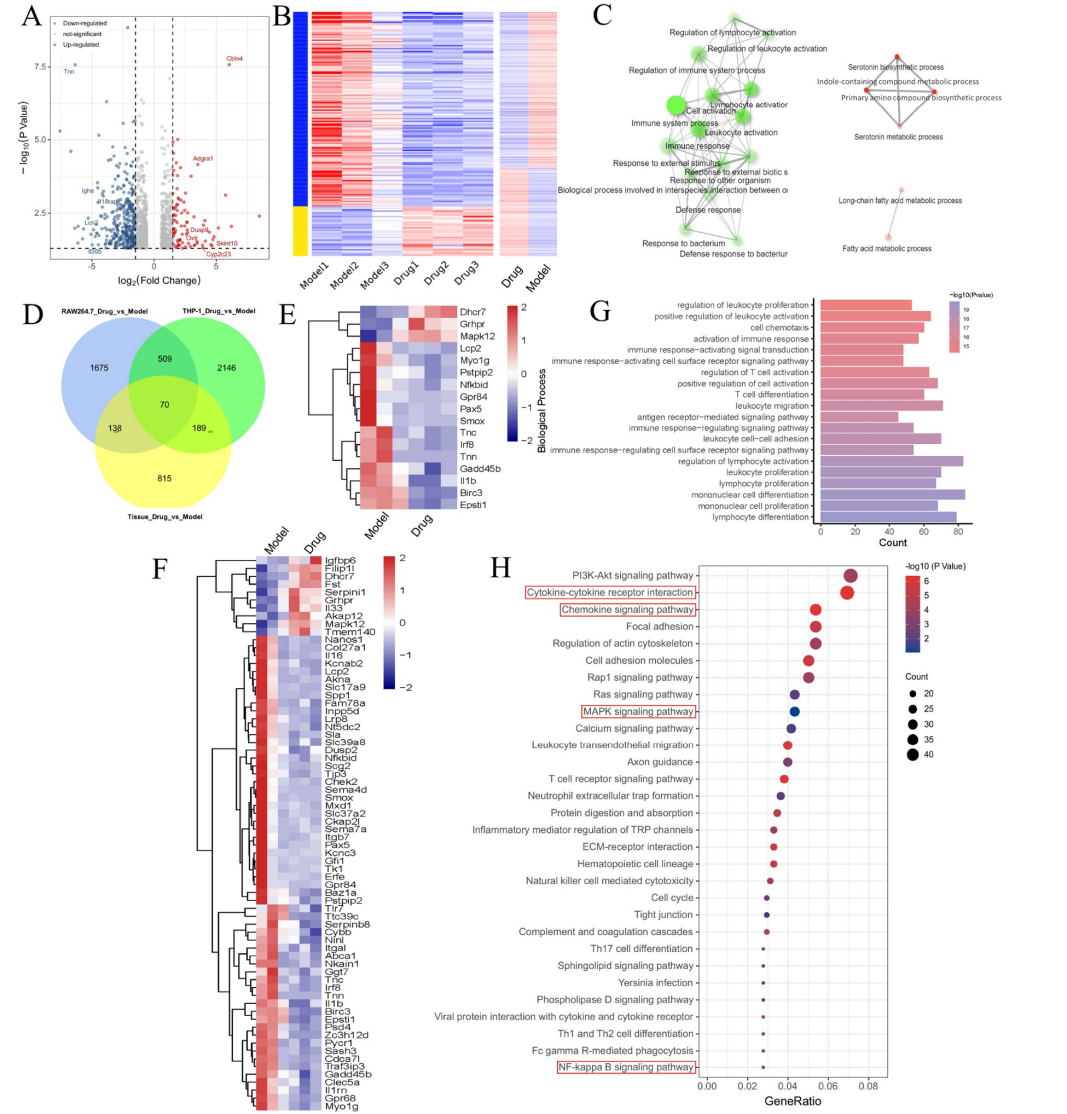
Figure 6 RNA-seq results of OXH in a CIA model (A) Compared with those in the CIA group, a total of 83 DEGs were upregulated and 355 DEGs were downregulated in the synovial tissue of the OXH group, as shown by a volcano plot of the mRNA expressions of genes mediating RA reversal. (B) Differential gene expression heatmaps of the CIA and RA groups and the top 2000 gene expression heatmaps of the two groups. (C) The network diagram of differential gene GO analysis; green indicates downregulation and red indicates upregulation. (D) The genes coexpressed between the CIA and RA groups among RAW264.7 macrophages, THP-1 cells and synovial tissues were identified by a Venn diagram. (E) Heatmap of the top 17 DEGs among the 70 DEGs. (F) Heatmap of the 70 coregulated DEGs between the CIA and RA groups in RAW264.7 macrophages, THP-1 cells and synovial tissues. (G) GO analysis of the CIA and OXH groups of RAW264.7 macrophages, THP-1 cells and synovial tissues. (H) KEGG enrichment analysis.

The underlying mechanism by which OXH alleviates RA in the CIA model was explored through transcriptome sequencing (RNA-seq). By adopting DESeq2, we selected DEGs on the basis of the thresholds of an absolute log fold change ≥1.5 and an adjusted *P* value<0.05. Compared with those in the CIA group, there were 83 upregulated and 355 downregulated DEGs in the OXH group, including genes related to inflammation. Notably, the downregulated genes included inflammatory cytokines *II36b*, *Ighe*, *Skint10*, *Ptgdr*, *Oxtr*, and *IL18rap*, which are related to activating and regulating MAPK and NIK/NF-κB. Additionally, Lcn2, an inflammatory cytokine, has emerged as a new marker for chronic inflammatory diseases such as RA. Dusp9 exhibits MAP kinase tyrosine phosphatase activity, while Adgra1 can activate G protein-coupled receptors associated with inflammation and pathological pain. These findings were accompanied by disturbances in downstream signaling pathways ( Figure 6A). A differential gene heatmap was generated to visualize the changes in the transcriptional levels of genes between the OXH and CIA groups, which indicated significant differential expression. Similarly, the top 2000 genes also exhibited significant changes ( Figure 6B). As revealed by GO analysis, the biological process (BP) terms involved were regulation of leukocyte activation, negative regulation of lymphocyte activation, positive regulation of serotonin biosynthesis, fatty acid metabolism, and long-chain fatty acid metabolism ( Figure 6C). Furthermore, pathways related to OXH for RA treatment were identified by KEGG pathway enrichment, and the top 30 pathways were identified. Notably, inflammation-related pathways, including the MAPK and NF-κB pathways, were among the most enriched pathways ( Figure 6H). The pathogenic mechanism of RA is related to the accumulation of immune cells around the synovial membrane, resulting in the production of inflammatory substances. Transcriptome analysis was conducted on LPS-induced RAW264.7 macrophages and PMA-stimulated THP-1 cells. In addition, the CIA group and OXH group were compared, which revealed 70 common DEGs in RAW264.7 macrophages, THP-1 cells, and synovial tissue ( Figure 6D). Additionally, a heatmap was generated to display the regulation of these 70 genes ( Figure 6F), and a subset of 17 genes with significant expression levels were selected for their role in regulating inflammation and immunity ( Figure 6E). Moreover, the top 20 GO terms were also enriched in immune-related biological processes ( Figure 6G). In summary, OXH alleviated RA primarily through the regulation of immune gene expression and the modulation of inflammatory factor production. This regulatory effect may involve downstream inflammatory cytokines and inflammatory mediators via the MAPK/NF-κB pathway and other pathways.

### OXH inhibited RA through the NF-κB/MAPK pathway

**Figure 7:**
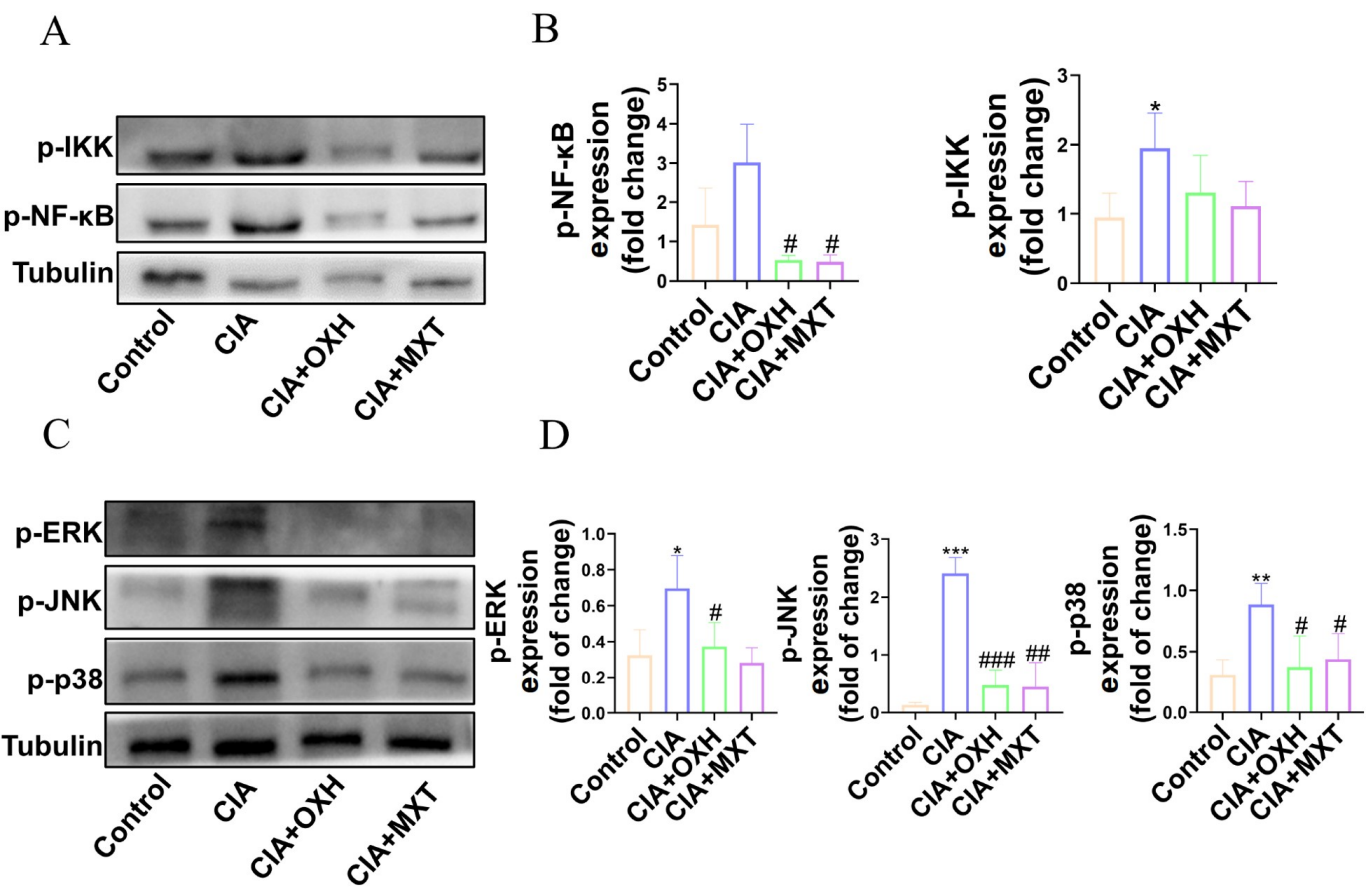
Figure 7 The effect of OXH on the NF-κB/MAPK pathway in the synovial tissue of CIA rats (A) Protein expressions of p-IKK and p-NF-κB in the synovial tissue of different treatment groups. (B) Quantitative statistical analysis of the protein expressions of p-IKK and p-NF-κB. (C) Protein expressions of p-JNK, p-ERK, and p-p38 in the synovial tissue of the different treatment groups. (D) Quantitative statistical analysis of the protein expressions of p-JNK, p-ERK, and p-p38. Data are expressed as the mean±SD. *P<0.05, **P<0.01, ***P<0.001, compared with the control group. #P<0.05, ## P<0.01, ###P<0.001, compared with the CIA group.

Notably, the NF-κB pathway plays a crucial role in regulating proinflammatory gene expression; therefore, different proinflammatory factors, such as TNF-α, IL-6, and IL-1β, are produced, thereby accelerating RA progression. Additionally, the MAPK pathway not only plays a critical role in regulating cytokine and metalloproteinase synthesis but is also responsible for regulating proinflammatory factors that contribute to cartilage destruction in RA [25]. Through transcriptome analysis, we confirmed the involvement of the NF-κB/MAPK pathway in the effect of OXH. Furthermore, p-NF-κB, p-IKK, p-p38, p-ERK and p-JNK protein levels in the synovial tissue of rats were markedly increased in the RA model group. However, after OXH treatment, the expressions of these proteins were decreased ( Figure 7A‒D). Therefore, OXH may have a therapeutic effect on RA by modulating the NF-κB/MAPK pathways.

## Discussion

RA is receiving increasing attention from people. Since the pathogenic mechanism of RA involves the secretion of various proinflammatory chemokines and factors, these substances can thus recruit and activate immune cells in the inflammatory microenvironment, leading to joint destruction. Although the drugs currently used in clinical practice have certain therapeutic effects, their high cost and accompanying side effects are becoming increasingly evident, creating an urgent need for new treatment options. Previous studies have reported that OXH suppresses TNF-α, IL-4, and IL-1β levels [26]. However, there are limited reports on whether OXH can effectively treat RA. In our study, compared with the model group, the OXH group exhibited significant improvements in body weight, immune organ function, and joint swelling ( Figure 4C,F,G). Additionally, CT and H&E staining demonstrated improved joint necrosis in the rats (Figures 4D and 5A). These findings provide preliminary evidence that OXH may attenuate RA symptoms. Furthermore, we attempted to verify these results using synovial cells (FLS-RAs), but unfortunately, the desired effect was not achieved.

As classical pathways, the NF-κB/MAPK pathway is crucial for the inflammatory response. MAPK regulates cellular responses to various activities and external stimuli, including inflammation, differentiation, apoptosis, and gene expression [ 27, 28]. MAPK-targeting inhibitors are synthesized for treating inflammatory diseases [29]. By affecting the NF-κB/MAPK signaling in inflammatory models, this pathway participates in the release of downstream inflammatory factors and mediators. Furthermore, suppressing ERK1/2, JNK1/2, and P38 expressions can prevent bone loss [27] .

Transcriptomic analysis is a valuable approach used to identify DEGs and signaling pathways. This analysis can provide valuable insights for drug toxicity assessment, drug efficacy evaluation, and drug combination therapy. Analysis of the RNA-Seq data ( Figure 6) revealed that OXH reversed the changes in the expressions of genes involved in inflammatory pathways, including the NF-κB pathway. In CIA rats, western blot analysis ( Figure 7) demonstrated that OXH inhibited the expressions of phosphorylated proteins, including p-IKK, p-NF-κB, p-JNK, p-ERK, and p-p38, thereby suppressing the downstream inflammatory factors IL-6, IL-1β, and TNF-α ( Figure 5). Furthermore, similar results were obtained in LPS-induced RAW264.7 macrophages, in which OXH reduced inflammation-induced ROS production and suppressed the levels of inflammatory mediators (iNOS and COX-2) and inflammatory factors (TNF-α, IL-6, and IL-1β) ( Figure 1). These effects were confirmed through the suppression of the upstream NF-κB/MAPK pathways ( Figure 2).

TLR4, a type I transmembrane receptor, activates the innate immune response by recognizing damage-related molecular patterns [30]. LPS is the outer surface component in gram-negative bacteria and acts as an exogenous agonist of TLR4 [31]. When it forms a complex dimer with TLR4, it activates intracellular adapter proteins and the intracellular supramolecular organizing center (SMOC) [ 32, 33]. This center then induces proinflammatory signaling cascades, thereby activating downstream NF-κB and MAPK-related pathways. To investigate the impact of OXH on downstream NF-κB and MAPK pathways through its interaction with TLR4 and LPS binding, we conducted CETSA experiments ( Figure 3A). The results revealed that OXH competed with LPS for simultaneous binding to the TLR4/MD2 complex. Furthermore, MST experiments demonstrated the distinctive affinity of OXH for the TLR4 protein, and the Kd value was 33.7 μM ( Figure 3B). As revealed by the molecular docking results, OXH bound to the pocket of TLR4/MD2 and interacted with several amino acids, such as GLY-343, LYS-388, PHE-345 and LYS-388, on another strand ( Figure 3C). The results of molecular dynamics simulations showed that OXH and TLR4/MD2 formed approximately four hydrogen bonds and had a stable binding capacity ( Figure 3D,E). This finding suggested that OXH directly interacts with the TLR4/MD2 complex and that subsequent allosteric regulation may contribute to the disruption of TLR4-related signaling pathways.

In conclusion, LPS-induced RAW264.7 macrophages were used for *in vitro* experiments, which also supported these findings, showing that OXH reduces inflammatory mediator and factor levels via the NF-κB/MAPK pathway. Additional analyses, including CETSA, MST, molecular docking and molecular dynamics simulations, revealed that OXH binds to the TLR4 active site, potentially inhibiting the interaction of TLR4/MD2 with LPS, thereby affecting the inflammatory response. The ability of OXH to attenuate RA was confirmed by using a CIA model *in vivo*. RNA-Seq data were analyzed to examine the related signaling pathways, especially the NF-κB and MAPK pathways, which were further validated at the protein level. These results confirmed that OXH alleviates RA through the NF-κB/MAPK pathway, consistent with the *in vitro* results. Overall, these findings highlight that OXH is a potential new therapeutic candidate for preventing and treating RA.
